# Supplementary material for: Research on germplasm diversity of Amomum villosum. Lour in genuine producing area
Source: PLoS One. 2022 Aug 31;17(8):e0268246. doi: 10.1371/journal.pone.0268246 (PMC9432772; doi:10.1371/journal.pone.0268246)
Supplement: S1 Raw images — (PDF) [file pone.0268246.s004.pdf]

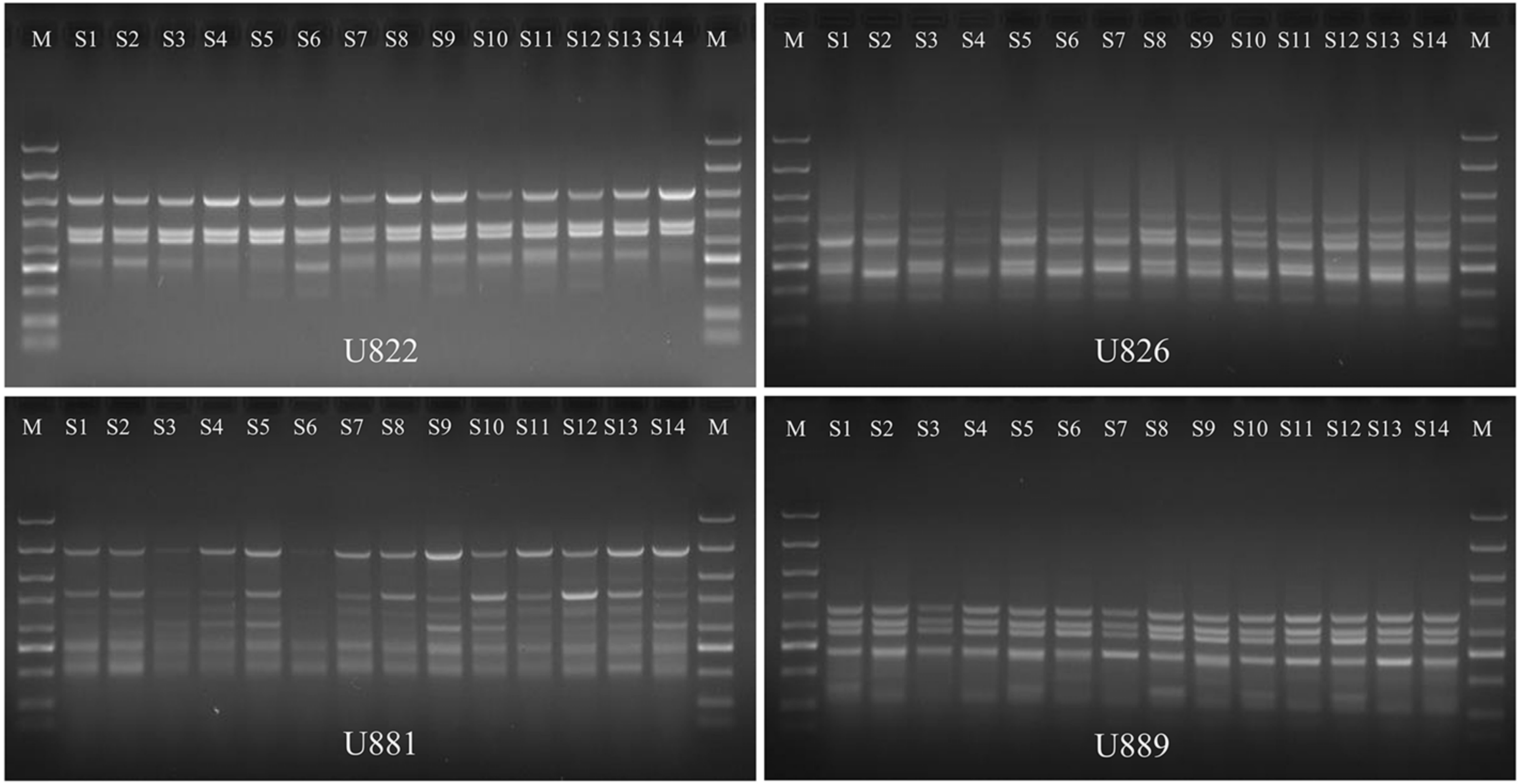

Fig 5. Amplification results of ISSR-PCR with different primers

This image is a combination of individual electrophoresis images of Fig 5(A-D), each electrophoresis image was taken with the Mshot MSX2 imaging system after 1.5 h of electrophoresis at 120 V in 1× TAE buffer and observation of the gel under UV light. The samples used were the RNA extracted from the *A. villosum* S1~S14 population in this study. The sample situation for each lane is shown in this figure

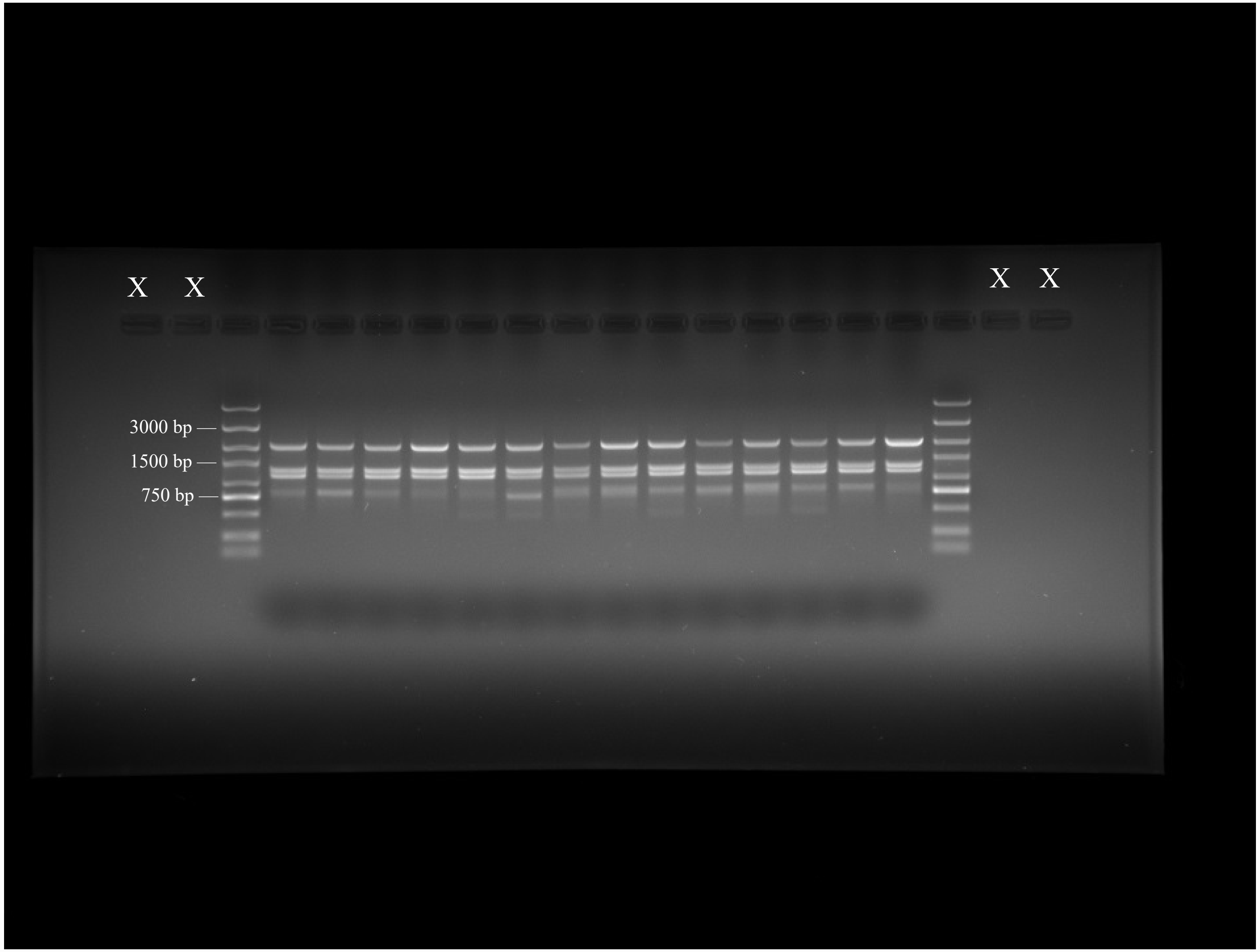

Fig 5(A). Primer 822 screening samples S1~14

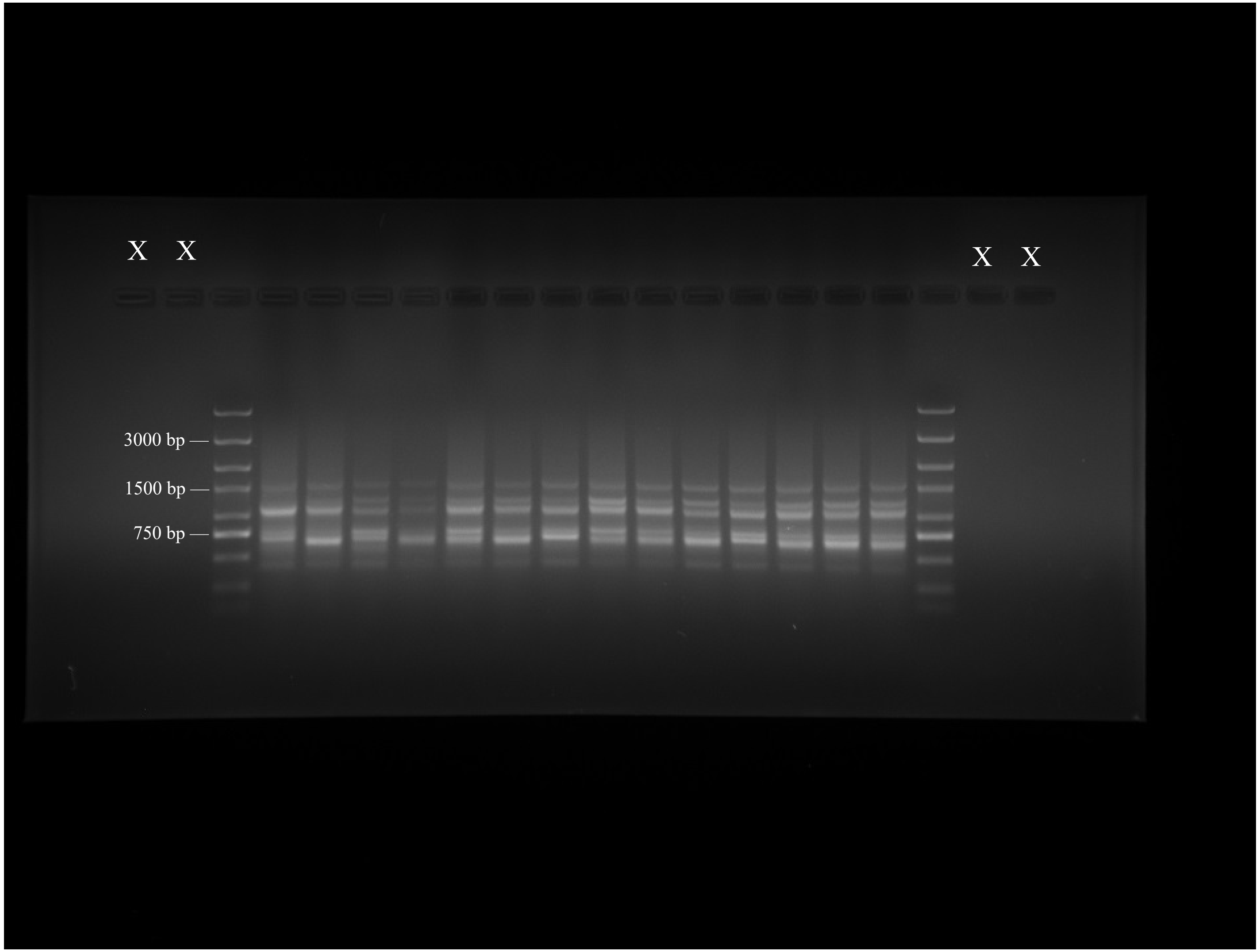

Fig 5(B). Primer 826 screening samples S1~14

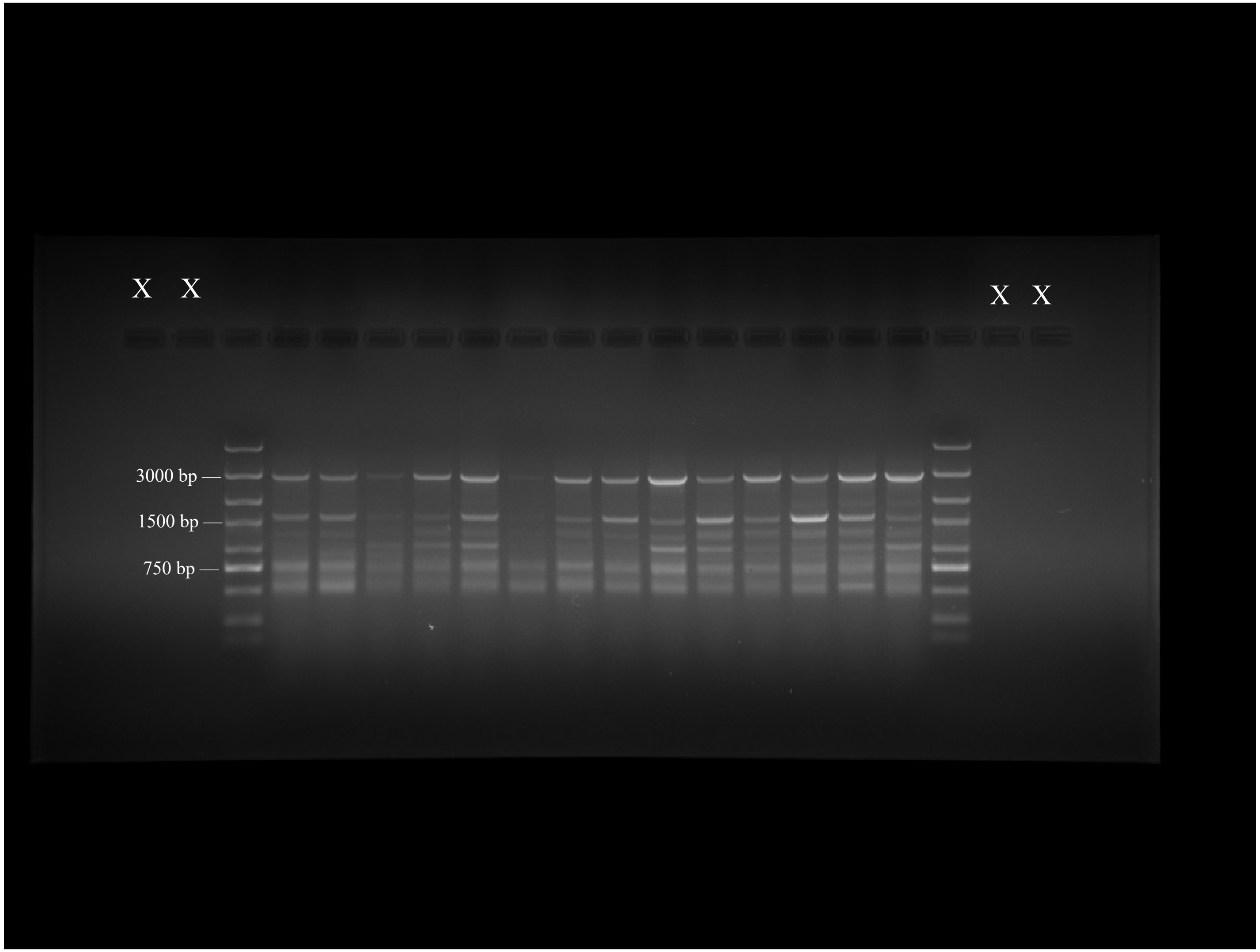

Fig 5(C). Primer 881 screening samples S1~14

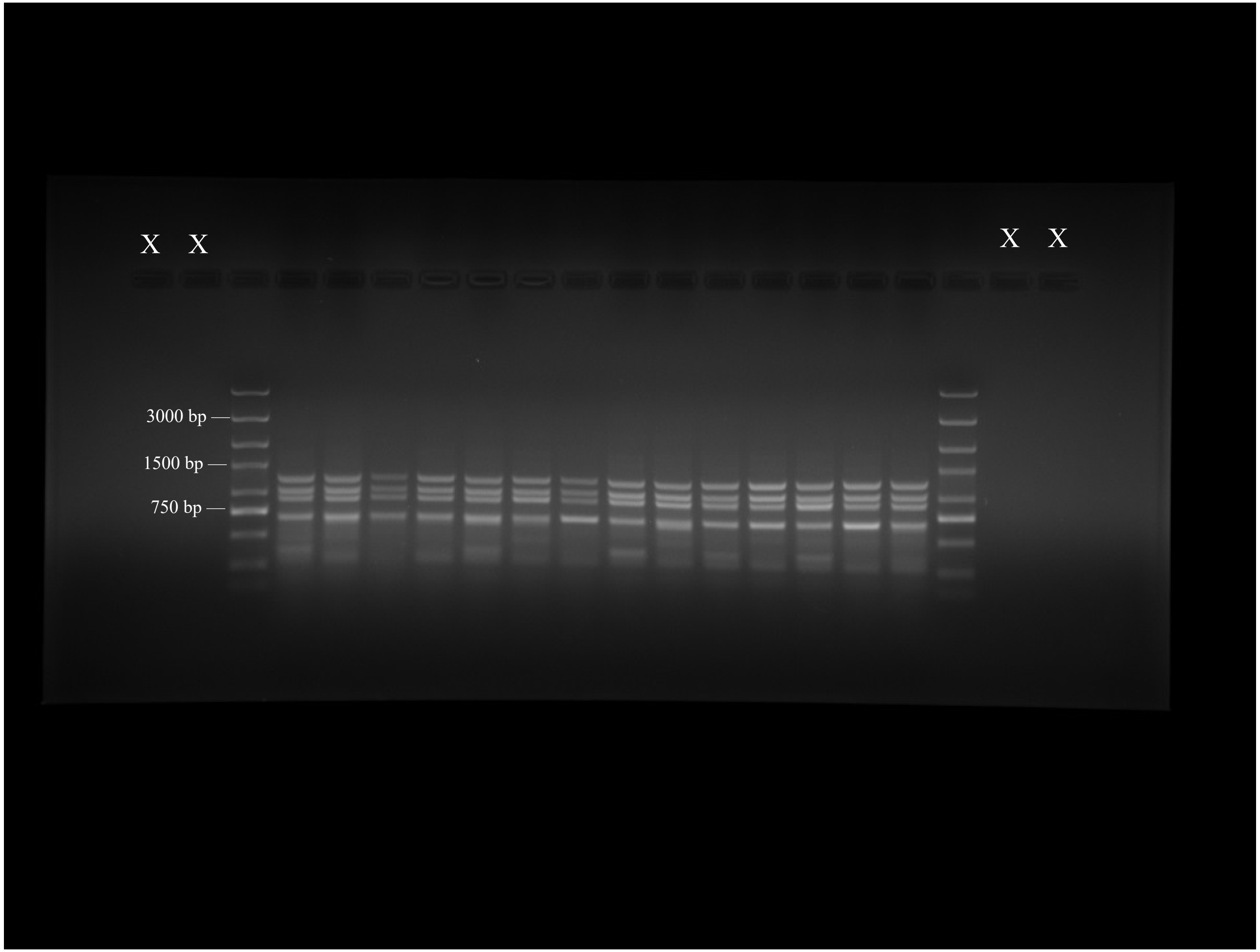

Fig 5(D). Primer 889 screening samples S1~14
