# Supplementary material for: Research on germplasm diversity of Amomum villosum. Lour in genuine producing area
Source: PLoS One. 2022 Aug 31;17(8):e0268246. doi: 10.1371/journal.pone.0268246 (PMC9432772; doi:10.1371/journal.pone.0268246)
Supplement: Attachment — Submitted filename: Response to Reviewers.docx [file pone.0268246.s005.docx]

Dear Reviewers,

Thanks for your interest in our manuscript entitled “Genetic Relationship and Quality Evaluation of *Amomum villosum*. Lour in Genuine Producing Area” (ID: PONE-D-22-00368).

We also appreciate your comments in the letter. The manuscript has been thoroughly revised based on your recommendations and that of the reviewers. However, we are willing to make further revisions if necessary.

Yours sincerely,

BoHan Yang

Manuscript Number: PONE-D-22-00368

PONE-D-22-00368
Genetic relationship and quality evaluation of Amomum villosum. Lour in genuine producing area
PLOS ONE

Dear Dr. He,

Thank you for submitting your manuscript to PLOS ONE. After careful consideration, we feel that it has merit but does not fully meet PLOS ONE’s publication criteria as it currently stands. Therefore, we invite you to submit a revised version of the manuscript that addresses the points raised during the review process.
Please submit your revised manuscript by Mar 31 2022 11:59PM. If you will need more time than this to complete your revisions, please reply to this message or contact the journal office at plosone@plos.org. When you're ready to submit your revision, log on to https://www.editorialmanager.com/pone/ and select the 'Submissions Needing Revision' folder to locate your manuscript file.

Please include the following items when submitting your revised manuscript:
A rebuttal letter that responds to each point raised by the academic editor and reviewer(s). You should upload this letter as a separate file labeled 'Response to Reviewers'.
A marked-up copy of your manuscript that highlights changes made to the original version. You should upload this as a separate file labeled 'Revised Manuscript with Track Changes'.
An unmarked version of your revised paper without tracked changes. You should upload this as a separate file labeled 'Manuscript'.
If you would like to make changes to your financial disclosure, please include your updated statement in your cover letter. Guidelines for resubmitting your figure files are available below the reviewer comments at the end of this letter.

If applicable, we recommend that you deposit your laboratory protocols in protocols.io to enhance the reproducibility of your results. Protocols.io assigns your protocol its own identifier (DOI) so that it can be cited independently in the future. For instructions see: <https://journals.plos.org/plosone/s/submission-guidelines#loc-laboratory-protocols>. Additionally, PLOS ONE offers an option for publishing peer-reviewed Lab Protocol articles, which describe protocols hosted on protocols.io. Read more information on sharing protocols at <https://plos.org/protocols?utm_medium=editorial-email&utm_source=authorletters&utm_campaign=protocols>.

We look forward to receiving your revised manuscript.

Kind regards,

Tzen-Yuh Chiang

Academic Editor

PLOS ONE

Journal Requirements:

When submitting your revision, we need you to address these additional requirements.

1. Please ensure that your manuscript meets PLOS ONE's style requirements, including those for file naming. The PLOS ONE style templates can be found at

https://journals.plos.org/plosone/s/file?id=wjVg/PLOSOne_formatting_sample_main_body.pdf and https://journals.plos.org/plosone/s/file?id=ba62/PLOSOne_formatting_sample_title_authors_affiliations.pdf

2. PLOS ONE now requires that authors provide the original uncropped and unadjusted images underlying all blot or gel results reported in a submission’s figures or Supporting Information files. This policy and the journal’s other requirements for blot/gel reporting and figure preparation are described in detail at https://journals.plos.org/plosone/s/figures#loc-blot-and-gel-reporting-requirements and https://journals.plos.org/plosone/s/figures#loc-preparing-figures-from-image-files. When you submit your revised manuscript, please ensure that your figures adhere fully to these guidelines and provide the original underlying images for all blot or gel data reported in your submission. See the following link for instructions on providing the original image data: https://journals.plos.org/plosone/s/figures#loc-original-images-for-blots-and-gels.

In your cover letter, please note whether your blot/gel image data are in Supporting Information or posted at a public data repository, provide the repository URL if relevant, and provide specific details as to which raw blot/gel images, if any, are not available. Email us at plosone@plos.org if you have any questions.

3. PLOS requires an ORCID iD for the corresponding author in Editorial Manager on papers submitted after December 6th, 2016. Please ensure that you have an ORCID iD and that it is validated in Editorial Manager. To do this, go to ‘Update my Information’ (in the upper left-hand corner of the main menu), and click on the Fetch/Validate link next to the ORCID field. This will take you to the ORCID site and allow you to create a new iD or authenticate a pre-existing iD in Editorial Manager. Please see the following video for instructions on linking an ORCID iD to your Editorial Manager account: https://www.youtube.com/watch?v=_xcclfuvtxQ

4. Please include captions for your Supporting Information files at the end of your manuscript, and update any in-text citations to match accordingly. Please see our Supporting Information guidelines for more information: http://journals.plos.org/plosone/s/supporting-information.

**Response to the editor:** We would like to thank you for considering the manuscript and for allowing the resubmission with major revision. We also thank the reviewers for their careful evaluation of our manuscript, and their detailed and thoughtful critiques. We have made extensive changes in the revised manuscript in accordance with the suggestions and requirements of the journal and the reviewers.

**Responses to Reviewers #1**

The article entitled "Genetic relationship and quality evaluation of Amomum villosum. Lour in genuine producing area" is well written and informative. Some major issues have been raised while reviewing the article:

1. Why did the authors focus only on bornyl acetates? By the GC-MS study?

**Response:** The standards for the content of bornyl acetate and volatile oil in fruits of *A. villosum* are specified in the 2020 edition of the Chinese Pharmacopoeia and are usually used to determine the quality and conformity of *A. villosum* fruits. Bornyl acetate in the volatile oil is the main component of the pharmacological effect of *A. villosum* fruits, and bornyl acetate accounts for the largest proportion of the volatile oil. Most of the current studies are conducted to determine the content of bornyl acetate by GC-MS method[1-7], therefore, this paper focuses on the content of bornyl acetate and determined by GC-MS.

2. Please provide the chromatogram picture for any S1.......... population.

**Response:** Thank you for your pertinent advice. The GC-MS chromatograms of each population have been supplemented as requested. Detailed information of GC-MS compounds, including compound structural formulas, RTs, peak areas, etc. are also supplemented in manuscript, as detailed in the revision.

3. Total number of metabolites: GC-MS studies have not been seen in the MS. Authors profile only six compounds. How ___________

**Response:** Thanks for the constructive comment. We determined the chemical composition in the volatile oil of *A. villosum* fruits from 14 populations by GS-MS and found that the relative amounts and corresponding peak areas in the chromatograms of the six compounds described in the manuscript were high, and that only these six compounds were detected in most of the germplasms. In addition, 11 compounds were detected in this GC-MS, but five of them were detected at low levels, two were detected in only one population, and only a few populations contained one or two of these five compounds, which were of low reference value, so only these six compounds were selected for the model analysis. The information about the five compounds with lower content were not put in the Table 3 of manuscript. However, we have uploaded the overall data regarding these 11 compounds as “Supporting information, S1 Table.” for your review. Corresponding GC-MS studies have been added in the revised manuscript.

4. Please proved the fruit pictures in results section

**Response:** Thank you for the valuable suggestions. Pictures of the corresponding fruit for each population have been provided as suggested, shown as “Figure 1” in the revised manuscript for details.

5. How many ISSR primers are used by the authors for ISSR? If there are four, why? (5th table)

**Response:** Thanks for the constructive comment. A total of 10 ISSR primers were selected for this study according to previous studies that used ISSR techniques to identify germplasm of *A. villosum*. Finally, we selected four primers with clear bands, obvious spacing and good polymorphism for this experiment [8-10].

6. The scale is missing in Fig 2. DPI of fig 3 must be increase. Add the percentage of similarity to Fig 4.

**Response:** Thank you for the helpful suggestion. Following your suggestion, figure 3 and figure 4 have been revised as requested, as detailed in the revised manuscript and uploaded attachments. Figure 2 was not changed because the scale was not indicated in the corresponding images in the existing studies involving HCA [11-15]. Since two new figures have been added to the revised draft, the numbers of the figures in the original manuscript have been changed accordingly, as detailed in the uploaded attachment.

7. Discussion part is poorly written in term of GC- MS profiling and ISSR. Kindly elaborate more with other studies.

**Response:** Thank you for the valuable suggestions. The discussion section has been reformulated with other GS-MS and ISSR-related studies as suggested, as detailed in the revised manuscript.

8. Kindly write the separate conclusions with translational values.

**Response:** Thank you for the valuable advice. We have added the "Conclusion" of this manuscript after the discussion, as detailed in lines 475 to 479 of the revised version.

9. I have only found molecular data to be novel and interesting. I would like to recommend a major revision of the MS. Authors must provide the details of compounds for GC-MS with peak area, RT, and total number of compounds in a separate table.

**Response:** Thank you very much for your interest in our manuscript and for your constructive comments and suggestions. Following your suggestion, we have provided details of the GC-MS compounds, including peak areas and retention times, in Table 3. Details of the total number of compounds in each population have been uploaded separately as “Supporting information, S1 Table.”. The manuscript has been thoroughly revised in accordance with your suggestions and we are willing to make further revisions if necessary.

**Responses to Reviewers #2**

**Reviewer #2:** Overall, this study focused on a topic of having both scientific and practical importance on development of *A. villosum*, a valuable herbal medicine. A comprehensive analysis on quality assessment as well as genetic diversity was performed. However, a strong concern about the present study might locate on its small germplasm size, which couldn’t well support a solid conclusion. Detailed comments are listed below.

**Response:** Thank you for your valuable and thoughtful comments. We will carefully incorporate them into our paper. All of your questions have been answered point-by-point.

**Major comments:**

1. The current title of the MS didn’t well fit its contents, since most of this study focused on quality evaluation rather than genetic relationship.

**Response:** Thanks for your constructive suggestion. The title has been changed as “Research on germplasm diversity of *Amomum villosum*. Lour in genuine producing area”.

Lines 2-3 (in the revision): “Research on germplasm diversity of *Amomum villosum*. Lour in genuine producing area”.

2. At the end of the Abstract, “Our data suggest that the germplasm of A. villosum is abundant in genuine producing area and that it is possible to select good germplasm resources in the area, expand the planting areas in non-genuine producing area and use a single germplasm resource for cultivation to obtain more quality products”, I can’t agree with that the results showed in Abstract could solidly and directly support its conclusion. I would suggest the author re-write the abstract, emphasizing on its highlight results clearly. Additionally, a brief background summary (e.g. introduction of genuine producing area) of the species is necessary in the beginning of Abstract section. Without background summary, readers might be confused about the results demonstrated in the Abstract.

**Response:** Thanks for your constructive suggestion. The section of the Abstract has been rewritten according to the corresponding recommendations, as detailed in lines 34 to 73 of revised manuscript.

3. Not only cultivation background but also research progresses of chemical components and genetic diversity of *A. villosum* should be briefly but clearly introduced in Introduction section.

**Response:** Thank you for the valuable suggestion. As per your suggestion, we have revised and written the section of the Introduction on the progress of research on the chemical composition and genetic diversity of *A. villosum*. Please see lines 83-89 and 96-103 in the revised version for detail.

4. Sampling information was incomplete. The author should add details of the sampling cite (latitude and longitude) when studying on genetic diversity analysis.

**Response:** Thank you for the valuable suggestion. As per your suggestion, detailed latitudinal and longitudinal information has been added to the samples collected for each population, as detailed in Table 1 in the revised manuscript.

5. The authors should clarify how many individuals they collected for each population. E.g. does S1 means one individual or several mixed samples from the same population? If yes, in areas 2,3&4, only one or two populations were collected for each area, resulting in a small germplasm size. Usually, a larger sample size would be acceptable in a genetic diversity study.

**Response:** Thanks for your constructive comment. Each population consists of many individuals, and we collected more than 20 fresh mature fruits and about 5 fresh leaves from different plants of each population as experimental samples. The fruits were used to measure biological traits, VOAV(volatile oil of *A. villosum*), BAAV(bornyl acetate of *A. villosum*), and the relative content of the major components of volatile oils, the leaves were used to extract RNA. The total number of individuals collected from each population has been clarified in Table 1 as required. Please see the revised manuscript for details.

6. “The fresh weight, dry weight, seed mass, seed mass dry weight, thousand-grain fresh weight (seed), and thousand-grain dry weight (seed) of each population of A. villosum dried fruit were measured by electronic balance”, here is a confusing expression, how did the author measure the fresh weight using dried fruits?

**Response:** Thank you for pointing out the mistake. We sincerely apologize for misleading you and we feel sorry about our carelessness. This expression is wrong. “*A. villosum* dried fruit” should be changed to “*A. villosum* fruits”, which has been corrected in line 189.

7. ISSR analysis in this MS seemed to be relatively independent. Results in this part didn’t show highlight significance to the whole study. Deeper discussions on genetic relationship with their quality evaluation would definitely make the study more valuable and interesting.

**Response:** Thank you for the valuable suggestion. As per your suggestion, we have discussed the genetic relationships of *A. villosum* and its quality assessment in more depth, taking an effort to make the manuscript more valuable and interesting for you and other readers. For details, please see lines 410-418 and 420-434 in the revised version.

8.  “The TOPSIS model was constructed based on the biological properties of the fruit and seed mass of A. villosum, VOAV and BAAV, and the relative content of the main components of the volatile oil”, based on what exact kind of biological properties did the authors determine as “good quality indicators? E.g. large seed mass size or high level of active component? The authors should explain in detail on the evaluation model parameters.

**Response:** Thanks for the constructive comment. In the TOPSIS model, the high superiority type index，“good quality indicators”, is an indicator that the higher the index value is, the better the germplasm is. In this paper, the biological characteristics of fruit and seed mass, the relative contents of VOAV and BAAV, and the main components of volatile oil are all "good quality indicators". For example, in *A. villosum* fruits the higher the relative contents of VOAV, BAAV and the main components of volatile oil there are, the better the quality of *A. villosum* fruits is. The greater the relative indexes of biological characteristics are, the greater the yield per unit area of *A. villosum* is and the higher income of growers have. The standardized decision matrix is an indispensable step in the TOPSIS model study, which is caused by the inconsistency of the magnitudes of the indicators in the multi-indicator decision analysis, so it is necessary to homogenize the data of the original decision matrix. The "Equation (1)" in the manuscript is applicable to the homogenization of the high-optimality type indicators[16-18].

9. “Third, after combining the comprehensive quality and genetic relationship analysis results of all samples, it is found that there are two or more different species of A. villosum in the same plot in the genuine producing area”, could the authors give the conclusion that S13&14 or S4&5 are different “species” based on evidences from the current study?

**Response:** Thanks for the constructive suggestion, but based on the available studies, it is not possible to conclude that S13&14 or S4&5 are different “species”, because species are the basic units of biological classification, natural groups of identical organisms that interbreed and segregate from other groups in reproduction. The breeding system of *A. villosum* is facultative autogamy, which means *A. villosum* can carry out selfing, and also outcrossing in some extent. S4&5 are from the same plot in Chunwan Town, while S13&S14 are from the same plot in Shaoguan City and were introduced from the same place of Chunwan, where the local growers rely on manual self-pollination or cross-pollination to produce the fruits of the *A. villosum*. Clearly, there is no reproductive segregate between S13&14 and S4&S5. In addition, the “variety” is a species that has some morphological variation, which is relatively stable. *A. villosum* Lour. var. *xanthioides* is a variety of the *A. villosum*, whose fruits are greenish in colour and the main component of the volatile oil is camphor, and the differences between the *A. villosum* Lour. var. *xanthioides* and the *A. villosum* are more stable. Further research could therefore identify S13&14 or S4&S5 as different “varieties” or not, but not S13&14 or S4&5 as different “species”[19].

**Minor comments**

1. Line and page numbers should be added to the MS for reviewers to give comments.

**Response:** Thank you for the helpful suggestion. Changes have been made in line as suggested, please see the revised version.

2. Full names should be presented when they first occurred in the MS. Revisions are needed in the Abstract and introduction sections, including but not limited to “A. villosum”, “ISSR”, “UPGMA”, “TOPSIS model”, and “RAPD”.

**Response:** Thank you for the helpful suggestion. Details of these abbreviations have been added as you requested, please see the revised version for details.

3. Minor changes are needed to suit grammar for the several sentences:
• The pharmacodynamic material basis of A. villosum is volatile oil, which mainly contains camphene, camphor, bornyl acetate and other chemical components, of which bornyl acetate is the most significant active component, which has pharmacological effects such as sedative and analgesic, anti-inflammatory and anti diarrhea, inhibition of fatty liver” and “but whether the diversity of germplasm is still abundant or not remains to be studied.
**Response:** Thank you for pointing out the grammatical mistake. We feel sorry about the improper expression in the sentences. We have rephrased the sentence, checked it several times for grammar and made it easy to understand. Please see lines 83-89 in the revision.

• The relative content of the main components of the volatile oil in each A. villosum population, the biological characteristics of the fruit and seed clusters, VOAV and BAAV were used as variables, combining with the orthogonal partial least squares discrimination analysis (OPLS-DA) and hierarchical clustering analysis (HCA) in the SIMCA 14.1 software to analyze the quality differences among 14 samples of A. villosum (Figure 1 & Figure 2).
**Response:** Thank you for pointing out the grammatical mistake. We feel sorry about the improper expression in the sentence. Please see lines 198-201 in the revised version for details of the changed sentence.

• The relative content of the main components of the volatile oil in each A. villosum population, the biological characteristics of the fruit and seed clusters, VOAV and BAAV were used as variables, combining with the orthogonal partial least squares discrimination analysis (OPLS-DA) and hierarchical clustering analysis (HCA) in the SIMCA 14.1 software to analyze the quality differences among 14 samples of A. villosum (Figure 1 & Figure 2).
**Response:** Thank you for pointing out the grammatical mistake. We feel sorry about the improper expression in the sentences. This sentence is a repetition of the previous sentence, and the question has been answered.

4. The authors should go though the whole MS for minor writing errors. E.g. in Introduction, “qi” should be “Qi”; In Material and methods section, “Traditional” should be “traditional”; A “,” is needed before “respectively”, etc.
**Response:** Thank you for pointing out these writing errors. We have read through the entire text and have double-checked and revised it based on your suggestions to make it clear and concise.

References

[1]  Chinese Pharmacopoeia Commission. Pharmacopoeia of the people's Republic of China. 1st ed. Beijing: China Medical Science Press, 2020, pp. 264-265.

[2]  Ao H, Wang J, Chen L, Li S, Dai C. Comparison of Volatile Oil between the Fruits of *Amomum villosum* Lour. and *Amomum villosum* Lour. var. *xanthioides* T. L. Wu et Senjen Based on GC-MS and Chemometric Techniques. Molecules. 2019; 24(9): 1663.

[3]  Cho JH, Lee JS, Kim HG, Lee HW, Fang Z, Kwon HH, et al. Ethyl Acetate Fraction of *Amomum villosum* var. *xanthioides* Attenuates Hepatic Endoplasmic Reticulum Stress-Induced Non-Alcoholic Steatohepatitis via Improvement of Antioxidant Capacities. Antioxidants (Basel). 2021; 10(7): 998.

[4]  Yin LJ, Wang YQ, Li P, Yang J, Wu JM, Tang C. Comparative analysis of fruit volatile oil composition of *Amomum villosum*. Lour and *Alpinia zerumbet*(Pers.) Burtt.et Smith by GC-MS. Journal of Chinese Medicinal Materials. 2020; 43(4): 891-895.

[5]  Xue X, Yang D, Wang D, Xu X, Zhu L, Zhao Z. Solidification of floating organic drop liquid-phase microextraction cell fishing with gas chromatography-mass spectrometry for screening bioactive components from *Amomum villosum* Lour. Biomedical chromatography. 2015; 29(4): 626-632.

[6]  Lu S, Zhang T, Gu W, Yang X, Lu J, Zhao R, et al. Volatile Oil of *Amomum villosum* Inhibits Nonalcoholic Fatty Liver Disease via the Gut-Liver Axis. BioMed research international. 2018; 2018: 3589874.

[7]  Li XG, Ye FQ, Xu HH. Study on pharmacological effects of bornyl acetate in volatile oil of *Amomum villosum.* West China Journal of Pharmaceutical Sciences. 2001; 16(5): 356-358.

[8]  Zhang ZL, Li XL, Yang CY, Tang DY, Wang JY, Gao WW. ISSR analysis on genetic diversity in *Amomi Fructus*. Chinese Traditional and Herbal Drugs. 2011; 42(3): 570-574.

[9]  Huang QL, Yang JF, Zhan RT, Yan P, Xu H, Chen WW. Molecular Identification of *Amomum villosum* Lour. Based on ISSR Analysis. Traditional Chinese Drug Research and Clinical Pharmacology. 2010; 21(5): 518-521.

[10] Gong L, Zhang DC, Ding XX, Huang J, Guan W, Qiu XH, et al. DNA barcode reference library construction and genetic diversity and structure analysis of *Amomum villosum* Lour. (Zingiberaceae) populations in Guangdong Province. Peer J. 2021; 9: e12325.

[11] Xu B, Wu C, Li Z, Song P, Chao Z. 1H NMR Combined with Multivariate Statistics for Discrimination of Female and Male Flower Buds of *Populus tomentosa*. Molecules. 2021; 26(21): 6458.

[12] Ma M, Wang T, Lu B. Assessment of genetic diversity in *Amomum tsao-ko* Crevost & Lemarié, an important medicine food homologous crop from Southwest China using SRAP and ISSR markers. Genetic resources and crop evolution. 2021; 68(6): 2655-2667.

[13] Ren R, Li Y, Chen H, et al. Carotenoid Contents of *Lycium barbarum*: A Novel QAMS Analyses, Geographical Origins Discriminant Evaluation, and Storage Stability Assessment. Molecules. 2021; 26(17): 5374.

[14] Wu C, Xu B, Li Z, Song P, Chao Z. Gender discrimination of *Populus tomentosa* barks by HPLC fingerprint combined with multivariate statistics. Plant Direction. 2021; 5(3): e00311.

[15] Zhang L, Wang X, Guo J, Xia Q, Zhao G, Zhou H, et al. Metabolic profiling of Chinese tobacco leaf of different geographical origins by GC-MS. Journal of agricultural and food chemistry. 2013; 61(11): 2597-2605.

[16] Zhuang X, Yin AT, Ren XG, Li W. A study on constructing a key index system for performance evaluation of general hospitals. Chinese Journal of Hospital Administration. 2006; 5: 341-344.

[17] Li Y, Zhang Q, Xu FR, Liu F, Wang YZ, Zhang JY. Multiple attribute decision making analysis TOPSIS on quality evaluation study of *Panax notoginseng*. Chinese Traditional and Herbal Drugs. 2017; 48(22): 4764-4771.

[18] Wang ZX, Li DD, Zheng HH. The External Performance Appraisal of China Energy Regulation: An Empirical Study Using a TOPSIS Method Based on Entropy Weight and Mahalanobis Distance. International journal of environmental research and public health. 2018; 15(2): 236.

[19] Zeng Z, Xi ZC, Meng SJ, Pang SM, Xie RQ, Ye XN, et al. Study on Volatile Constitutions and Quality Evaluation of Different Varieties of Fructus Amomis. Journal of Instrumental Analysis. 2010; 29(7): 701-706.
