# Supplementary material for: Research on germplasm diversity of Amomum villosum. Lour in genuine producing area
Source: PLoS One. 2022 Aug 31;17(8):e0268246. doi: 10.1371/journal.pone.0268246 (PMC9432772; doi:10.1371/journal.pone.0268246)
Supplement: Attachment — Submitted filename: Response to Reviewers.docx [file pone.0268246.s006.docx]

PONE-D-22-00368R1

Research on germplasm diversity of Amomum villosum. Lour in genuine producing area

PLOS ONE

Dear Dr. He,

Thank you for submitting your manuscript to PLOS ONE. After careful consideration, we feel that it has merit but does not fully meet PLOS ONE’s publication criteria as it currently stands. Therefore, we invite you to submit a revised version of the manuscript that addresses the points raised during the review process.

Please submit your revised manuscript by Jun 02 2022 11:59PM. If you will need more time than this to complete your revisions, please reply to this message or contact the journal office at [plosone@plos.org](mailto:plosone@plos.org). When you're ready to submit your revision, log on to https://www.editorialmanager.com/pone/ and select the 'Submissions Needing Revision' folder to locate your manuscript file.

Please include the following items when submitting your revised manuscript:

- A rebuttal letter that responds to each point raised by the academic editor and reviewer(s). You should upload this letter as a separate file labeled 'Response to Reviewers'.
- A marked-up copy of your manuscript that highlights changes made to the original version. You should upload this as a separate file labeled 'Revised Manuscript with Track Changes'.
- An unmarked version of your revised paper without tracked changes. You should upload this as a separate file labeled 'Manuscript'.

If you would like to make changes to your financial disclosure, please include your updated statement in your cover letter. Guidelines for resubmitting your figure files are available below the reviewer comments at the end of this letter.

If applicable, we recommend that you deposit your laboratory protocols in protocols.io to enhance the reproducibility of your results. Protocols.io assigns your protocol its own identifier (DOI) so that it can be cited independently in the future. For instructions see: <https://journals.plos.org/plosone/s/submission-guidelines#loc-laboratory-protocols>. Additionally, PLOS ONE offers an option for publishing peer-reviewed Lab Protocol articles, which describe protocols hosted on protocols.io. Read more information on sharing protocols at <https://plos.org/protocols?utm_medium=editorial-email&utm_source=authorletters&utm_campaign=protocols>.

We look forward to receiving your revised manuscript.

Kind regards,

Tzen-Yuh Chiang
Academic Editor
PLOS ONE

Journal Requirements:

Please review your reference list to ensure that it is complete and correct. If you have cited papers that have been retracted, please include the rationale for doing so in the manuscript text, or remove these references and replace them with relevant current references. Any changes to the reference list should be mentioned in the rebuttal letter that accompanies your revised manuscript. If you need to cite a retracted article, indicate the article’s retracted status in the References list and also include a citation and full reference for the retraction notice.

Please use the space provided to explain your answers to the questions above. You may also include additional comments for the author, including concerns about dual publication, research ethics, or publication ethics. (Please upload your review as an attachment if it exceeds 20,000 characters)

**Response to the editor:** We would like to thank you for considering the manuscript and for allowing the resubmission with minor revision. We also thank the reviewers for their careful evaluation of our manuscript, and their thoughtful critiques. We have revised the manuscript in accordance with the suggestions and requirements of the journal and the reviewers, and have carefully checked the references. In addition, to meet graduation needs, we have readjusted the ranking of the co-first authors (top two authors) after consultation with the corresponding and other authors of this manuscript, but the contribution of the co-first authors remains unchanged and all authors have agreed to this adjustment.

**Responses to Reviewers #1**

Reviewer #1: Dear Authors please add some important points in the revised MS.

Minor comments
previous Q.

How many ISSR primers are used by the authors for ISSR? If there are four, why? (5th table)

Response: Thanks for the constructive comment. A total of 10 ISSR primers were selected for this study according to previous studies that used ISSR techniques to identify germplasm of A. villosum. Finally, we selected four primers with clear bands, obvious spacing and good polymorphism for this experiment [8][10].

New suggestions: Response in MS: Twenty-three selected ISSR primers were used in the germplasm identification study of A. villosum2930, line number 242-243, Please re-check and correct in the MS.

**Response:** Thank you for pointing out the mistake. We sincerely apologize for misleading you and we feel sorry about our carelessness. We have revised this error here, see the revised version for details.


Q. Discussion part is poorly written in term of GC- MS profiling and ISSR. Kindly elaborate more with other studies.
Response: Thank you for the valuable suggestions. The discussion section has been reformulated with other GS-MS and ISSR-related studies as suggested, as detailed in the revised manuscript.

New suggestions-Authors improved some part of this section, but I will suggest kindly elaborate more with previous study.

**Response:** Thank you for the valuable advice. Following your suggestion, we have added some points about variations of the *A. villosum* in the “Discussion”. For details, please see lines 358-364 in the revised vision.

Conclusions: Please add some points related to chemotypes and its variations (GC-MS study).

**Response:** Thank you very much for your valuable and thoughtful comments. In response to your suggestion, we have added relevant content to the revised manuscript. However, the chemotypes and variations of *A. villosum* have been less well studied by GC-MS techniques. Apologies that no more information could be provided.

**Responses to Reviewers #2**

Reviewer #2: The MS has been much improved in the current vision, and most of the comments have been addressed. Several minor comments are listed below.
1. Line 14, “consistent quality” should be “consistently qualified”.
2. Line 21-24, move “In this study, we selected 12 populations… for quality evaluation and genetic variation detection” to “methods” section.
3. Line 339&345, pay attention to the reference number format errors.

**Response:** Thank you for pointing out the error of grammar and errors in reference format. We feel sorry about the inappropriate expression and incorrect format in the sentences. We have carefully checked and revised the grammar and the format of the reference in the revised manuscript. In addition, as per your suggestion, lines 21-24 of the original draft have been moved to “Material and Methods”. Please see lines 99-103 in the revision.
